# Effects of Dietary Metabolizable Energy and Crude Protein Levels on Growth Performance, Carcass Traits, and Meat Quality of Goslings from 35 to 63 Days of Age  

**DOI:** 10.3390/foods15061060

**Published:** 2026-03-17

**Authors:** Xuan Li, Xucheng Zheng, Xiyuan Xing, Wenfeng Liu, Qingxue Liu, Zhi Yang, Haiming Yang, Zhiyue Wang

**Affiliations:** 1 The Animal Science and Technology College, Yangzhou University, Yangzhou 255300, China; liooo1111@163.com (X.L.); dx120230175@stu.yzu.edu.cn (X.Z.); mz120231558@stu.yzu.edu.cn (X.X.); holdon30mins@gmail.com (W.L.); lqx0215lqx@163.com (Q.L.); hmyang@yzu.edu.cn (H.Y.); 2 Joint International Research Laboratory of Agriculture and Agri-Product Safety, Ministry of Education of China, Yangzhou University, Yangzhou 225009, China; zhiyang@yzu.edu.cn

**Keywords:** goslings, low-protein diet, production performance, meat color

## Abstract

Dietary metabolizable energy (ME) and crude protein (CP) are key determinants of production efficiency in geese; however, their combined effects during the rapid growth phase are not well defined. A total of 240 male goslings were assigned to four treatments in a 2 × 2 factorial arrangement, with six replicates per treatment and 10 birds per replicate. We used a 2 × 2 factorial design to evaluate two ME levels (11.20 vs. 11.65 MJ/kg) and two CP levels (16% vs. 14%) in goslings from 35 to 63 days of age. Growth performance, carcass traits, meat quality, serum biochemical indices, and instrumental taste attributes were measured. Increasing ME increased body weight at day 63 and average daily gain (*p* < 0.05), whereas average daily feed intake and feed-to-gain ratio were not affected. Most carcass traits were unchanged; however, leg muscle percentage differed between ME levels (*p* < 0.01) and was higher in the 11.20 MJ/kg group. Meat color responses were muscle- and time-dependent: breast b* at 45 min postmortem was affected by ME and CP (*p* < 0.001), and leg color traits at 45 min exhibited significant ME × CP interactions (*p* < 0.05). Postmortem pH, water-holding capacity, and shear force were largely unaffected by dietary treatments. Serum glucose showed a significant ME × CP interaction (*p* = 0.001), and triglyceride concentration was influenced by both ME and CP (*p* < 0.01), with lower values observed at higher ME and lower CP. Instrumental taste attributes did not differ among treatments (*p* > 0.05). In conclusion, modest changes in dietary ME and CP modulated growth and selected carcass, color, and metabolic traits without compromising key technological meat-quality parameters. These results indicate that, during 35–63 days of age, the higher-ME diet (11.65 MJ/kg) combined with a moderate CP reduction to 14% can be considered a feasible formulation option under the conditions of this study.

## 1. Introduction

Goose production is expanding as consumers increasingly value goose meat as a nutrient-dense poultry product. Goose meat is a rich source of high-quality protein, B vitamins (e.g., vitamin B12), and essential minerals such as iron and zinc [1]. Global goose meat production reached ~4.4 million tons in 2022, ranking fourth among poultry species worldwide [1]. Despite this market potential, profitability in intensive goose production is highly sensitive to dietary cost because feed is one of the largest inputs in poultry systems and can account for up to ~70% of variable production costs [2]. The 35–63-day period (≈5–9 weeks) is commonly targeted for nutritional interventions in growing geese [3], coinciding with accelerated body and muscle accretion and continued maturation of the digestive tract around 5–6 weeks of age [4,5]. During this stage, muscle growth shows pronounced temporal dynamics (e.g., rapid leg muscle accretion from 15 to 49 days and rapid breast muscle accretion after 42 days) [5], implying a high and time-sensitive nutrient demand to support efficient tissue deposition. In this context, dietary metabolizable energy (ME) and crude protein (CP) are two core nutritional drivers of growth and tissue accretion. Because energy supply constrains the efficiency with which amino acids are utilized for lean tissue deposition, dietary ME should be considered together with CP and amino acid balance rather than in isolation [6,7]. Accordingly, precise balancing of dietary energy and protein during the rapid-growth phase represents a key nutritional lever for improving growth efficiency and nutrient utilization in geese.

Dietary ME is a major determinant of growth and nutrient partitioning in poultry because adequate energy supply underpins efficient utilization of other nutrients for tissue accretion [7]. Birds generally regulate feed intake to meet energy requirements; thus, increasing dietary ME typically reduces voluntary intake, with downstream consequences for feed efficiency and body composition. In geese, a quantitative synthesis of 16 studies conducted during 5–10 weeks of age reported a negative association between ME and average daily feed intake (ADFI) and suggested an apparent optimal ME range (~12–13 MJ/kg) for improving feed efficiency, while also indicating a tendency toward increased abdominal fat deposition within this range [6]. These findings underscore a trade-off between efficiency gains and excessive fat accretion under high-energy feeding.

Crude protein supplies amino acids required for rapid muscle accretion and is therefore a key driver of growth performance and feed efficiency. In growing geese, inappropriate protein supply—whether deficient or excessive—can impair efficiency and increase nitrogen excretion, with both economic and environmental consequences [8]. Beyond performance, excessive protein intake may also increase health risks in goslings; high-protein diets have been associated with cecal dysbiosis and kidney injury linked to gout development [9]. Reduced-CP strategies are increasingly explored to mitigate nitrogen emissions; however, lowering CP can compromise feed conversion and promote fat deposition when amino acid supply becomes limiting [10]. Consistent with this trade-off, a layer study reported that reducing CP improved protein utilization and decreased nitrogen excretion but impaired production performance, highlighting the context dependence of reduced-CP outcomes [11]. In geese, moderate CP reduction may be feasible under specific dietary conditions, indicating that potential benefits are conditional rather than universal [12]. Importantly, the effectiveness of any CP strategy is inherently dependent on the dietary energy context.

Metabolizable energy and CP do not act independently; growth responses reflect their dynamic balance because energy availability governs the efficiency with which dietary amino acids are converted into body protein [7]. Empirical studies have reported ME × CP interactions, suggesting that the optimal ME level depends on concurrent protein supply and the overall dietary energy-to-protein ratio [13,14]. When CP is reduced without corresponding adjustments in energy density and amino acid adequacy, nutrient partitioning may shift toward fat deposition, and feed efficiency may deteriorate [10]. Nevertheless, systematic evidence defining optimal ME–CP combinations for geese during the rapid growth phase remains limited. Therefore, factorial experiments designed to disentangle their interactive effects are needed to establish evidence-based recommendations for energy–protein balance in growing geese.

Despite the recognized importance of ME and CP in poultry nutrition, most research has focused on broilers or ducks, whereas evidence for geese—particularly during the late growing period—is comparatively scarce [13,15]. Moreover, existing studies have often evaluated ME or CP independently, with limited attention to their interactive effects on carcass composition, postmortem meat-quality dynamics, and metabolic responses. Given that geese exhibit distinct growth trajectories, muscle fiber characteristics, and fat deposition patterns relative to other poultry species, dietary energy–protein balance may exert species-specific effects on tissue accretion and meat quality development. Comprehensive assessments that integrate growth performance, carcass traits, conventional meat-quality indices, serum biochemical parameters, and instrumental taste attributes within a single factorial design remain uncommon. Accordingly, elucidating the combined effects of dietary ME and CP in growing goslings is necessary to refine nutritional strategies that enhance production efficiency while maintaining desirable meat quality. However, integrative evaluations that simultaneously link growth performance, carcass traits, conventional meat-quality indices, serum biochemistry, and instrumental taste attributes in a factorial design remain uncommon. Therefore, the present study used a 2 × 2 factorial arrangement to test two ME levels and two CP levels in male Jiangnan White goslings from 35 to 63 d of age, with the aim of characterizing main effects and ME × CP interactions on performance, carcass composition, early postmortem color traits, metabolic indicators, and instrumental taste attributes.

## 2. Materials and Methods

All animal procedures were approved by the Institutional Animal Care and Use Committee of Yangzhou University (approval No.: 202403131). As the animal sampling protocols had received prior approval for commercial implementation, no additional licensing was required. Throughout the study, humane techniques, including rapid cervical dislocation, were employed. All procedures were carried out with consistent care to minimize animal discomfort and in strict compliance with animal welfare and ethical standards.

### 2.1. Experimental Design and Diets

At 35 days of age, 240 male goslings were randomly allocated to four treatment groups based on body weight, with six replicate pens per treatment and 10 birds per pen; the pen (replicate) was considered the experimental unit for growth performance, whereas one bird per pen was selected for slaughter and laboratory measurements and treated as the experimental unit for carcass traits, serum biochemistry, and meat-quality analyses.

A completely randomized 2 × 2 factorial design was employed, consisting of two dietary metabolizable energy (ME) levels (11.20 MJ/kg, normal; and 11.65 MJ/kg, high) and two crude protein (CP) levels (16%, normal; and 14%, low). The four dietary treatments were as follows: (1) NPNE (16% CP, 11.20 MJ/kg ME), (2) LPNE (14% CP, 11.20 MJ/kg ME), (3) NPHE (16% CP, 11.65 MJ/kg ME), and (4) LPHE (14% CP, 11.65 MJ/kg ME).

Diet formulation was based on the nutrient requirements for geese recommended by the NRC (1994), combined with previous research experience of the Poultry Production and Nutrition Laboratory of Yangzhou University [12,16]. Corn and soybean meal were used as the main ingredients. The ingredient composition and calculated nutrient levels of the experimental diets are presented in Table 1.

### 2.2. Animal and Housing

A total of 240 healthy 35-day-old male Jiangnan White goslings were used in the present study at the Yangzhou University Gaoyou Campus, Gaoyou, Jiangsu, China. Prior to the start of the experiment, the poultry house, drinkers, and feeders were thoroughly cleaned, rinsed, and disinfected. The experiment was conducted in an indoor floor-pen house with a closed structure and mechanical ventilation, where each pen measured 1.90 m × 1.50 m (2.85 m^2^), housed 10 birds, and provided ad libitum access to feed (mash form) and water. The ambient temperature was maintained at 22–28 °C, and a lighting schedule of approximately 16 hours of light per day was applied. It consisted of natural daylight supplemented with 2 h of artificial lighting.

### 2.3. Growth Performance and Sample Collection

During the experiment, the total amount of feed offered and the residual feed were recorded to calculate feed intake. Body weight was measured after fasting at 35 days of age (baseline) and 63 days of age (end of experiment). Average daily feed intake (ADFI), average daily gain (ADG), and feed-to-gain ratio (F/G; ADFI/ADG) were calculated accordingly.

At 63 days of age, after fasting and final body weight measurement, one gosling with body weight close to the replicate mean was randomly selected from each replicate for blood collection and slaughter. The birds were humanely euthanized strictly in accordance with the AVMA Guidelines for the Euthanasia of Animals (2020 Edition). Specifically, the geese were rendered unconscious via an electrical water bath stunning (Parameters: 65 V, 86 mA, 400 Hz for 18 s per bird) to ensure immediate insensibility [18].

Carcass weight, eviscerated weight, semi-eviscerated weight, breast muscle weight, leg muscle weight, intestinal fat weight, and abdominal fat weight were recorded. The semi-eviscerated weight was calculated by removing the trachea, esophagus, intestines, spleen, pancreas, gallbladder, reproductive organs, and gizzard contents and corneum from the carcass. The eviscerated weight was calculated by removing the heart, liver, proventriculus, gizzard, lungs, and abdominal fat from the semi-eviscerated carcass. The carcass, semi-eviscerated carcass, and eviscerated carcass percentages were calculated relative to the live BW before slaughter. The breast muscle, leg muscle, and abdominal fat percentages were calculated relative to the eviscerated weight.

### 2.4. Serum Biochemical Analysis

On day 63, after a feed withdrawal of 6 h, blood samples were collected from the wing vein of one bird per replicate pen into sterile vacuum procoagulant tubes. Samples were allowed to clot at room temperature for 30–60 min, and were then centrifuged at 3500 r/min for 15 min. Serum was carefully aspirated, aliquoted into 1.5 mL microcentrifuge tubes, and stored at −20 °C until analysis (avoiding repeated freeze–thaw cycles).

Serum concentrations of total protein (TP), albumin (ALB), globulin (GLB), glucose (GLU), total cholesterol (TC), triglycerides (TG), high-density lipoprotein cholesterol (HDL-c), low-density lipoprotein cholesterol (LDL-c), urea, and creatinine were determined using an automatic biochemical analyzer (Hitachi 7600, Tokyo, Japan) with corresponding commercial reagent kits according to the manufacturer’s instructions [19]. The albumin-to-globulin ratio (A/G) was calculated as ALB/GLB, and globulin was obtained as GLB = TP − ALB. All samples were analyzed in the same laboratory run when possible, and internal quality controls provided with the analyzer were used to verify assay performance.

### 2.5. Meat Quality

The right breast and right leg muscles were collected for pH determination at 45 min and 24 h postmortem. After sampling at 45 min, the muscle samples designated for the 24 h measurements were stored at 4 °C until analysis. The left breast and left leg muscles were used for measurements of color parameters, cooking loss, drip loss, shear force, and taste-related characteristics; these samples were placed on ice immediately after collection and analyzed promptly (measured immediately after sampling).

Meat color values, including lightness (L*), redness (a*), and yellowness (b*), as well as water loss rate, were determined according to Determination of Meat Quality in Livestock and Poultry (NY/T 1333-2007) [20]. Briefly , L*, a*, and b* were measured using a colorimeter (CR 400, Konica Minolta, Tokyo, Japan), with th-ree readings per sample and the mean value recorded. Muscle pH at 45 min and 24 h postmortem (samples stored at 4 °C for the 24 h measurement) was determined using a pH meter (pH-STAR, Matthäus, München, Germany), with three measurements per sample averaged. Shear force was measured following Determination of Meat Tenderness—Shear Force Method (NY/T 1180-2006) [21]. For cooking loss, trimmed breast and leg muscle samples (fascia and visible fat removed) were weighed, sealed in zip-lock bags, and heated in an 80 °C water bath until the geometric center reached 70 °C; samples were then blotted dry and reweighed to calculate cooking loss as (pre-cooking weight − post-cooking weight)/pre-cooking weight × 100%. The cooked samples were subsequently cut into three strips (1 × 1 × 2.5 cm), and shear force was determined using a tenderness tester (RH-N50, Runhu, Guangzhou, China), with the mean value used for analysis.

The taste attributes of goose breast muscle (sourness, bitterness, astringency, bitter aftertaste, astringent aftertaste, umami, richness, saltiness, and sweetness) were measured using an electronic tongue system (SA402B, Intelligent Sensor Technology Co., Ltd., Atsugi, Japan) [22].

### 2.6. Statistical Analysis

Data were analyzed using SPSS 22.0 (IBM, Armonk, NY, USA). Normality of the original data was assessed prior to analysis, and all variables met the normality assumption. Homogeneity of variance was evaluated using Levene’s test. Growth performance variables were analyzed with the pen (replicate) as the experimental unit, whereas carcass traits, serum biochemical indices, meat quality, and instrumental taste attributes were analyzed with the individual bird as the experimental unit (one bird per replicate). A general linear model (GLM) was applied to perform two-way ANOVA, with dietary metabolizable energy level (ME) and crude protein level (CP) as fixed effects, including the ME × CP interaction. When a significant interaction was detected, comparisons among the four treatment combinations (NPNE, LPNE, NPHE, and LPHE) were conducted using Fisher’s least significant difference (LSD) test. Results are presented as mean ± SEM. Differences were considered significant at *p* < 0.05, and 0.05 ≤ *p* < 0.10 was considered a statistical trend. Radar charts were prepared using Origin 2026 (OriginLab Corporation, Northampton, MA, USA).

## 3. Results

### 3.1. Growth Performance

Growth performance from 35 to 63 days of age is shown in Table 2. Goslings receiving the higher ME diet (11.65 MJ/kg) reached a greater final body weight than those fed 11.20 MJ/kg (*p* < 0.05). A similar pattern was observed for ADG, which was higher under the 11.65 MJ/kg ME level (*p* < 0.05). By contrast, ADFI and F/G remained comparable between ME levels and were also not influenced by CP level (*p* > 0.05). No interaction was detected for any growth trait (*p* > 0.05).

### 3.2. Slaughter Performance

Carcass traits measured at 63 days of age are summarized in Table 3. Dressing percentage and eviscerated yield did not differ among treatments (*p* > 0.05). Leg muscle yield, however, showed a clear response to dietary ME (*p* < 0.05), with a higher leg muscle percentage observed in goslings fed 11.20 MJ/kg compared with 11.65 MJ/kg. Breast muscle yield and abdominal fat percentage were not significantly altered by either ME or CP (*p* > 0.05). In addition, ME × CP interactions were not evident for carcass indices (*p* > 0.05).

### 3.3. Meat Quality

#### 3.3.1. Meat Color Traits

Breast and leg muscle color parameters measured at 45 min and 24 h postmortem are presented in Table 4 and Table 5.

For breast muscle, b* at 45 min differed between ME levels and between CP levels (*p* < 0.05), whereas L* and a* at 45 min were similar across treatments (*p* > 0.05). Specifically, b* at 45 min was higher at 11.65 MJ/kg than at 11.20 MJ/kg and was also higher in the 14% CP group compared with the 16% CP group. At 24 h, b* remained associated with CP (*p* < 0.05), with higher values in the 14% CP group, while the effect of ME was not significant (*p* < 0.05). No ME × CP interaction was detected for breast muscle color traits at either time point (*p* > 0.05).

For leg muscle, dietary ME significantly affected color parameters at 45 min postmortem (L*, a*, and b*; *p* < 0.05). Specifically, compared with 11.20 MJ/kg, the higher ME level (11.65 MJ/kg) resulted in lower L* but higher a* and higher b* at 45 min. At 24 h, ME influenced a* (*p* < 0.05), whereas L* and b* did not differ between ME levels (*p* > 0.05). CP affected L* at 45 min (*p* < 0.05) and b* at 24 h (*p* < 0.05). Moreover, significant ME × CP interactions were detected for leg muscle L*, a*, and b* at 45 min (*p* < 0.05), indicating that early postmortem color development in leg muscle was shaped by the combined dietary ME and CP levels.

#### 3.3.2. pH at 45 Min and 24 h

Muscle pH values at 45 min and 24 h postmortem are presented in Table 6. Neither ME nor CP altered pH decline patterns in breast or leg muscle (*p* > 0.05). Water-holding capacity–related traits (cooking loss and drip loss) and shear force are shown in Table 7. These indices were generally stable across treatments, and no significant main effects or interactions were detected (*p* > 0.05).

### 3.4. Serum Biochemical Indices

Serum biochemical indices are presented in Table 8. Among the measured parameters, GLU and TG were responsive to dietary treatments (*p* < 0.05). GLU showed a significant ME × CP interaction (*p* < 0.05), with the highest value in LPNE and the lowest value in LPHE. TG was affected by ME and CP as main effects (ME: *p* < 0.05; CP: *p* < 0.05), showing lower TG at the higher ME level and under the lower CP level; correspondingly, NPNE exhibited the highest TG, and LPHE the lowest TG. ALB showed a CP main effect (*p* < 0.05), whereas TP, GLB, A/G, TC, HDL-C, LDL-C, and urea were not significantly affected (*p* > 0.05).

### 3.5. Taste-Related Traits

**Figure 1:**
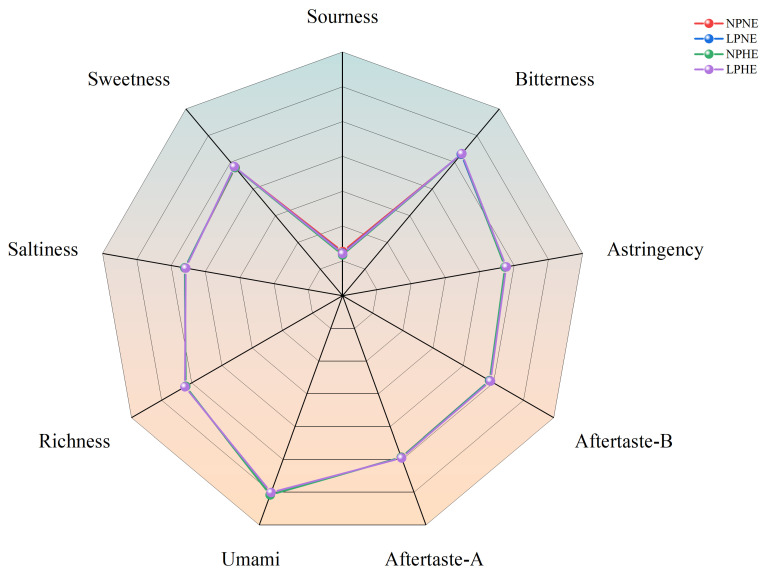
Effects of dietary ME and CP levels on taste attributes of gosling meat at 63 d of age.

Taste-related indices are shown in Table 9 and Figure 1. No significant effects of ME, CP, or their interaction were detected (*p* > 0.05), suggesting that the instrumental taste profile was not sensitive to the tested dietary treatments.

## 4. Discussion

This study employed a 2 × 2 factorial design to examine the main effects of dietary metabolizable energy (ME) and crude protein (CP), as well as their interaction, on growth performance, carcass traits, meat quality, and circulating metabolic indices in male Jiangnan White geese from 35 to 63 days of age. Under the conditions tested, the nutritional implication was generally consistent: increasing ME from 11.20 to 11.65 MJ/kg enhanced growth, as indicated by higher body weight at 63 days and greater average daily gain, whereas reducing CP from 16% to 14% did not compromise growth performance. Importantly, these dietary manipulations were not reflected in broad changes in conventional meat-quality traits; instead, responses were first detected in more sensitive endpoints, including muscle color parameters and serum metabolites. This contrast suggests that practical adjustments in the energy–protein supply may modify metabolic status before producing measurable changes in structural meat-quality traits.

A notable observation was that the higher-ME diet improved growth without significantly altering average daily feed intake (ADFI) or the feed-to-gain ratio (F/G). This pattern is informative because it implies that the performance advantage was not primarily intake-driven. Rather, a plausible interpretation is that increasing dietary energy density increased net energy availability per unit of feed and thereby supported faster tissue accretion. Accordingly, the results are more consistent with improved energetic support and nutrient partitioning than with behavioral regulation of voluntary intake. This interpretation aligns with poultry nutrition evidence showing that moderate increases in ME, within practical formulation limits, can translate into growth advantages even when feed intake remains relatively stable [23,24,25]. Studies in waterfowl likewise indicate that dietary ME can reshape growth trajectories and carcass development, although responses vary with production phase and nutritional background [14,26,27,28]. For example, Zeng et al. reported significant ME × CP interactions in Pekin ducks (15–35 d), where body weight gain responses to protein became more pronounced as dietary ME increased, and optimal BWG/FCR occurred under the high-AMEn plus high-CP combination, underscoring the importance of matching energy supply to protein deposition demands [26]. In growing Pekin ducks (15–42 d), Wu et al. found that increasing dietary ME (10.82 to 12.95 MJ/kg) reduced ADFI and improved feed conversion, while average daily gain remained comparatively stable, indicating that efficiency gains can occur without proportional increases in intake [27]. Similarly, in Pekin ducks (21–38 d), synchronously increasing nutrient density (higher ME and CP) improved BWG and feed efficiency while decreasing feed intake linearly, suggesting that higher dietary energy can support growth with reduced voluntary consumption under certain production settings [28].

More broadly, ME rarely functions as an isolated driver of performance. The biological benefit of additional dietary energy depends on whether protein and amino acid nutrition are sufficient to sustain lean tissue deposition. Consequently, the dietary energy-to-protein balance is often the primary determinant of growth efficiency, rather than ME or CP considered independently [24,29,30]. Within this framework, the response to higher ME in the present study is best interpreted as improved energetic support for accretion across the tested CP range, rather than a nonspecific “high-energy advantage.”

Reducing CP from 16% to 14% did not impair growth performance (*p* > 0.05), supporting the inference that, within this age window and dietary context, a moderate reduction in CP can be implemented without overt performance penalties. Consistent with this, Belloir et al. reported that, when amino acid profiles were adjusted, reducing broiler dietary CP by ~2–3 percentage points could be achieved without compromising body weight gain or feed intake [31]. Similarly, Min et al. observed in goslings that BW gain at 17.5% CP was comparable to that at 20.0% CP, and both exceeded 15.0% CP, supporting the feasibility of moderate CP reduction when nutritional adequacy is maintained [32]. Critically, the key factor is not crude protein per se, but whether the amino acid supply remains functionally adequate and avoids limitations to lean deposition. This principle underpins reduced-CP strategies in poultry, where the objective is precision nutrition—maintaining amino acid adequacy while reducing unnecessary nitrogen input—rather than indiscriminate protein reduction [31,33,34]. Evidence from geese and ducks further indicates that protein responses depend on the magnitude of CP reduction, amino acid balance, and accompanying energy supply [14,28,32,35]. Thus, the present findings support feasibility while emphasizing that “reduced CP” should be treated as a formulation strategy with explicit boundary conditions.

A key carcass outcome was the lower leg muscle percentage under the higher-ME diet. Because this variable is expressed as a proportion, the decline more likely reflects a redistribution of carcass components than a true suppression of leg muscle accretion. In practical terms, higher dietary energy may have promoted deposition of other tissues or depots (e.g., breast muscle and/or adipose fractions), thereby increasing the denominator against which leg yield is expressed and diluting the leg proportion. This interpretation is consistent with poultry evidence that dietary manipulation can shift cut yields and carcass composition without necessarily producing large changes in overall carcass weight or total yield [25,30]. In addition, leg and breast muscles may differ in metabolic and endocrine sensitivity to dietary cues, potentially contributing to divergent regional deposition patterns [36]. To confirm this explanation and clarify mechanisms, future studies should report absolute cut weights (g) alongside proportional yields and directly quantify fat depots (e.g., subcutaneous, intermuscular, and abdominal fat).

Across dietary treatments, pH at 45 min and 24 h, water-holding capacity, and shear force did not differ (*p* > 0.05), indicating that the ME and CP adjustments tested did not materially affect major postmortem processes related to acidification, water retention, or texture. The stability of pH (early and ultimate), WHC, and shear force indicates resilience of core postmortem physicochemical processes under moderate ME–CP optimization, consistent with the notion that these traits are less sensitive than color or blood metabolites within a narrow formulation range. Similar findings have been reported in poultry, where diet-induced shifts in metabolism or performance do not necessarily translate into changes in core physicochemical meat-quality traits [28,31,37]. In contrast, color traits were more responsive. Breast muscle b* at 45 min postmortem was affected by both ME and CP, and CP remained significant for b* at 24 h. In the leg muscle, a significant ME × CP interaction was detected, indicating that the color response depended on the combined energy–protein supply. Because early postmortem color is closely linked to pigment chemistry and oxygen-related reactions, it may respond to dietary background even when pH, water-holding capacity, and shear force remain unchanged. Reports that ME and CP can influence muscle composition and metabolic status [36], together with waterfowl studies showing context-dependent meat-quality responses to energy–protein strategies [26,28]. support this interpretation. Overall, within the formulation range evaluated, the observed color shifts likely reflect dietary sensitivity rather than clear deterioration of meat quality. Similarly, instrumental taste traits were unchanged, implying that the present dietary adjustments did not markedly shift the pool of water-soluble taste-active compounds; this may reflect limited changes in muscle free amino acids and nucleotides under these conditions.

Serum biochemical indices provided an informative system-level layer for interpreting dietary effects beyond performance and carcass outcomes. The significant ME × CP interaction for glucose supports the concept that glucose homeostasis is governed by energy–protein balance rather than by either nutrient alone [29,34]. Triglycerides were influenced by both ME and CP, suggesting that lipid handling is nutritionally adjustable even when gross carcass fat-related traits appear stable. Comparable patterns—where circulating metabolites respond earlier than overt carcass outcomes—have been reported under protein manipulation or nutrient-structure interventions in poultry [38,39]. Digestive utilization may contribute to these systemic signals; studies in ducks indicate that dietary ME and CP can modulate digestive enzyme activities, providing a plausible mechanistic link between diet formulation and downstream metabolic readouts mechanistic bridge between formulation design and downstream metabolic readouts [27,35]. Although digestive physiology markers were not measured here, incorporating them would help disentangle effects on digestion/absorption from post-absorptive nutrient partitioning in future work.

The ME × CP interaction observed for serum glucose is biologically consistent with coupled protein–energy metabolism [40]. In growing birds, the metabolic payoff of additional dietary energy depends on whether the amino acid supply is sufficient to sustain net protein deposition. When energy–protein supply is mismatched, a greater fraction of amino acids may be catabolized for ATP production or routed into gluconeogenic processes after deamination, thereby shifting glucose homeostasis and reducing the efficiency of lean accretion [41]. Conversely, when dietary energy is adequate and amino acid supply remains functionally sufficient, amino acids are preferentially retained for deposition while glucose is spared for essential tissues, consistent with a more protein-sparing metabolic state [42]. Under this framework, an ME increment would not be expected to exert a uniform effect across CP levels; rather, its metabolic consequence can differ depending on whether CP/AA supply places the animal in a deposition-favorable versus amino-acid–limited state. The concurrent sensitivity of triglycerides to ME and CP further supports that lipid handling is nutritionally adjustable within the present formulation window, consistent with hepatic re-partitioning of substrates among oxidation, lipogenesis, and lipid export before overt carcass fatness becomes detectable.

From an applied perspective, the findings support a practical formulation message for this growth window: increasing ME from 11.20 to 11.65 MJ/kg improved BW at 63 days, and ADG, and reducing CP from 16% to 14% was tolerated without compromising major performance indices or core physicochemical meat-quality traits. This outcome is consistent with efforts to optimize energy–protein balance and implement reduced-CP strategies that maintain production efficiency while improving nitrogen utilization and sustainability [31,33,34]. Nevertheless, these inferences are specific to the 35–63-day period and the formulation range tested. Because nutrient requirements and partitioning change with age, validation across production stages would strengthen generalizability and translational relevance [14,32].

Collectively, the present findings suggest that modest adjustment of dietary energy density can enhance growth during 35–63 days, whereas a moderate reduction in CP within this formulation window can be implemented without compromising overall performance or key technological meat-quality traits. Importantly, the nutrition signal was expressed more clearly in metabolically sensitive endpoints (e.g., circulating metabolites and early postmortem color traits) than in conventional texture- or water-holding–related indices, implying that metabolic partitioning may shift before structural quality traits are measurably altered. These results highlight the importance of considering ME and CP as a coupled nutritional system as they provide a practical basis for optimizing energy–protein balance in geese under intensive production conditions. A limitation of this study should be acknowledged. The experiment was conducted only during the 35–63-day growth phase, and the findings may not be directly applicable to other age periods or production stages.

## 5. Conclusions

Within the tested formulation range, goslings receiving 11.65 MJ/kg dietary metabolizable energy exhibited superior growth performance compared with those fed 11.20 MJ/kg during 35–63 days of age, whereas reducing crude protein from 16% to 14% did not compromise overall performance. Key technological meat-quality traits—including pH at 45 min and 24 h postmortem, water-holding capacity, and shear force—were maintained across dietary treatments. Together with diet-associated shifts in metabolic indices (e.g., serum glucose), these results indicate that modest ME–CP optimization can modulate metabolic status without adversely affecting major meat-quality endpoints. From a practical standpoint, a slightly higher-ME diet coupled with a moderate reduction in CP represents a feasible, cost-oriented formulation strategy for goslings during this growth phase.

## Figures and Tables

**Table 1:** Ingredient composition and nutrient levels of experimental diets (air-dry basis).

Ingredients (%)	NPNE	LPNE	NPHE	LPHE
Corn (CP, 8.0%)	61.85	65.30	56.05	59.65
Soybean meal (CP, 44.3%)	23.60	17.44	23.70	17.70
Wheat bran (CP, 14.3%)	5.64	7.92	8.50	10.39
Rice husk	5.29	5.48	5.13	5.41
Limestone	1.05	1.08	1.05	1.08
Dicalcium phosphate	1.11	1.12	1.10	1.10
DL-Methionine	0.16	0.20	0.17	0.21
L-Lysine	0.00	0.16	0.00	0.16
Salt	0.30	0.30	0.30	0.30
Soybean oil	0.00	0.00	3.00	3.00
Premix ^1^	1.00	1.00	1.00	1.00
Total	100.00	100.00	100.00	100.00
nutrient levels				
Metabolizable energy (MJ/kg)	11.22	11.20	11.65	11.65
Crude protein (%)	16.01	14.06	16.02	14.01
Crude fiber (%)	5.49	5.44	5.50	5.46
Lysine (%)	0.83	0.83	0.83	0.83
Methionine (%)	0.43	0.43	0.43	0.43
Calcium (%)	0.82	0.82	0.82	0.82
Available phosphorus (%)	0.35	0.35	0.35	0.35

Notes: NPNE, normal-protein normal-energy diet; LPNE, low-protein normal-energy diet; NPHE, normal-protein high-energy diet; LPHE, low-protein high-energy diet. ^1^ Premix provided per kg of diet: vitamin A, 9000 IU; vitamin D3, 4000 IU; vitamin E, 25 mg; vitamin K3, 1.5 mg; vitamin B1, 0.9 mg; vitamin B2, 8 mg; vitamin B6, 3.2 mg; vitamin B12, 0.01 mg; niacin, 45 mg; pantothenic acid, 11 mg; folic acid, 0.65 mg; biotin, 0.05 mg; choline chloride,350 mg; Fe, 60 mg; Cu, 10 mg; Mn, 95 mg; Zn, 90 mg; I, 0.5 mg; Se, 0.3 mg. Crude protein and crude fiber in the nutrient level are measured values, and the rest are calculated values. Crude protein was determined using the Kjeldahl method. Metabolizable energy values were calculated based on ingredient composition tables (China Feed Database, 2024) and formulated using standard prediction equations [17].

**Table 2:** Effects of dietary metabolizable energy (ME) and crude protein (CP) levels on growth performance of goslings from 35 to 63 d of age.

Items	ME (MJ/kg)	CP (%)	BW at 63 d (g)	ADG (g/d)	ADFI (g/d)	F/G
NPNE	11.20	16	3804.67	60.12	284.80	4.74
LPNE		14	3865.00	62.18	282.84	4.55
NPHE	11.65	16	3922.00	64.06	288.26	4.51
LPHE		14	3882.67	62.73	286.47	4.57
ME	11.20		3834.83 ^b^	61.15 ^b^	283.82	4.65
	11.65		3902.33 ^a^	63.39 ^a^	287.36	4.54
CP		16	3863.33	62.09	286.53	4.63
		14	3873.83	62.46	284.65	4.56
SEM			20.298	0.725	1.703	0.048
*p*-value	ME		0.029	0.041	0.157	0.141
	CP		0.718	0.721	0.444	0.357
	Interaction		0.098	0.115	0.971	0.079

NPNE, normal-protein normal-energy; LPNE, low-protein normal-energy; NPHE, normal-protein high-energy; LPHE, low-protein high-energy. ME, metabolizable energy; CP, crude protein; BW, body weight; ADG, average daily gain; ADFI, average daily feed intake; F/G, feed-to-gain ratio; SEM, standard error of the mean. Different superscript letters indicate significant differences between ME levels (*p* < 0.05).

**Table 3:** Effects of dietary ME and CP levels on carcass traits and meat yield of goslings at 63 d of age.

Items	ME (MJ/kg)	CP (%)	Dress. (%)	Evisc. (%)	Semi-Evisc. (%)	Breast Yield (%)	Leg Yield (%)	Abd. Fat (%)
NPNE	11.20	16	86.72	73.99	82.91	7.81	13.48	3.69
LPNE		14	87.00	73.91	82.77	7.50	14.70	3.33
NPHE	11.65	16	87.09	74.32	83.43	7.49	12.34	3.96
LPHE		14	87.39	75.07	84.50	7.00	12.63	3.84
ME	11.20		86.86	73.95	82.84	7.65	14.09 ^a^	3.51
	11.65		87.24	74.70	83.97	7.25	12.48 ^b^	3.90
CP		16	86.91	74.15	83.17	7.65	12.91	3.83
		14	87.20	74.49	83.64	7.25	13.66	3.58
SEM			0.256	0.509	0.483	0.190	0.322	0.155
*p* value	ME		0.490	0.311	0.114	0.146	0.002	0.096
	CP		0.597	0.647	0.504	0.156	0.113	0.283
	ME × CP		0.982	0.572	0.386	0.745	0.525	0.588

NPNE, normal-protein normal-energy; LPNE, low-protein normal-energy; NPHE, normal-protein high-energy; LPHE, low-protein high-energy. ME, metabolizable energy; CP, crude protein; Dress., dressing percentage; evisc., eviscerated yield; semi-evisc., semi-eviscerated yield; breast, breast muscle percentage; leg, leg muscle percentage; abdominal fat, abdominal fat percentage. SEM, standard error of the mean. Different superscript letters indicate significant differences between ME levels (*p* < 0.05).

**Table 4:** Effects of dietary ME and CP levels on breast muscle color parameters of goslings at 63 d of age.

Items	ME (MJ/kg)	CP (%)	45 min			24 h		
			L*	a*	b*	L*	a*	b*
NPNE	11.20	16	45.44	12.61	2.91	44.51	18.90	7.45
LPNE		14	46.03	12.40	4.36	43.84	19.35	8.19
NPHE	11.65	16	44.86	12.62	3.85	45.22	18.76	6.90
LPHE		14	45.28	11.97	4.82	43.38	19.45	7.70
ME	11.20		45.73	12.51	3.63 ^b^	44.17	19.12	7.82
	11.65		45.07	12.30	4.34 ^a^	44.30	19.10	7.30
CP		16	45.15	12.62	3.38 ^b^	44.86	18.83	7.17 ^b^
		14	45.66	12.18	4.59 ^a^	43.61	19.40	7.95 ^a^
SEM			0.457	0.175	0.159	0.466	0.252	0.168
*p* value	ME		0.497	0.560	<0.001	0.894	0.966	0.092
	CP		0.604	0.239	<0.001	0.203	0.293	0.016
	ME × CP		0.925	0.535	0.062	0.548	0.819	0.912

NPNE, normal-protein normal-energy; LPNE, low-protein normal-energy; NPHE, normal-protein high-energy; LPHE, low-protein high-energy. ME, metabolizable energy; CP, crude protein; L*, lightness; a*, redness; b*, yellowness. SEM, standard error of the mean. Means within a column with different superscript letters differ (*p* < 0.05).

**Table 5:** Effects of dietary ME and CP levels on leg muscle color parameters of goslings at 63 d of age.

Items	ME (MJ/kg)	CP (%)	45 min			24 h		
			L*	a*	b*	L*	a*	b*
NPNE	11.20	16	48.20	11.51	4.34	39.48	16.77	8.58
LPNE		14	43.35	14.03	6.09	40.45	17.35	11.14
NPHE	11.65	16	43.33	14.54	6.58	40.40	17.80	8.85
LPHE		14	43.72	12.95	4.83	38.74	18.64	9.80
ME	11.20		45.77 ^a^	12.77 ^b^	5.21 ^b^	39.96	17.06 ^b^	9.86
	11.65		43.53 ^b^	13.74 ^a^	5.71 ^a^	39.57	18.22 ^a^	9.32
CP		16	45.76 ^a^	13.03	5.46	39.94	17.29	8.71 ^b^
		14	43.54 ^b^	13.49	5.46	39.60	17.99	10.47 ^a^
SEM			0.635	0.301	0.218	0.428	0.271	0.290
*p* value	ME		0.037	0.020	0.044	0.652	0.029	0.227
	CP		0.039	0.242	0.990	0.695	0.171	0.001
	ME × CP		0.017	<0.001	<0.001	0.141	0.795	0.075

L*, lightness; a*, redness; b*, yellowness. SEM, standard error of the mean. Means within a column with different superscript letters differ (*p* < 0.05).

**Table 6:** Effects of dietary ME and CP levels on breast and leg muscle pH at 45 min and 24 h postmortem in goslings at 63 d of age.

Items	ME (MJ/kg)	CP (%)	Breast Muscle		Leg Muscle	
			45 min	24 h	45 min	24 h
NPNE	11.20	16	6.57	5.82	6.65	5.91
LPNE		14	6.53	5.86	6.65	5.92
NPHE	11.65	16	6.63	5.83	6.67	5.90
LPHE		14	6.62	5.84	6.66	5.88
ME	11.20		6.55	5.84	6.65	5.91
	11.65		6.62	5.84	6.67	5.89
CP		16	6.60	5.83	6.66	5.90
		14	6.57	5.85	6.66	5.90
SEM			0.027	0.012	0.010	0.009
*p* value	ME		0.058	0.856	0.177	0.084
	CP		0.477	0.190	0.776	0.842
	ME × CP		0.673	0.500	0.571	0.442

NPNE, normal-protein normal-energy; LPNE, low-protein normal-energy; NPHE, normal-protein high-energy; LPHE, low-protein high-energy. ME, metabolizable energy; CP, crude protein; pH was measured at 45 min and 24 h postmortem. SEM, standard error of the mean.

**Table 7:** Effects of dietary ME and CP levels on breast muscle quality traits of goslings at 63 d of age.

Items	ME (MJ/kg)	CP (%)	Cooking Loss (%)	Drip Loss (%)	Shear Force (N)
NPNE	11.20	16	25.86	4.30	61.92
LPNE		14	25.70	4.41	61.70
NPHE	11.65	16	25.31	4.39	60.54
LPHE		14	25.79	4.38	61.07
ME	11.20		25.78	4.35	61.81
	11.65		25.55	4.39	60.80
CP		16	25.58	4.34	61.23
		14	25.75	4.40	61.39
SEM			0.159	0.104	0.511
*p* value	ME		0.502	0.889	0.356
	CP		0.630	0.819	0.883
	ME × CP		0.348	0.796	0.730

NPNE, normal-protein normal-energy; LPNE, low-protein normal-energy; NPHE, normal-protein high-energy; LPHE, low-protein high-energy. ME, metabolizable energy; CP, crude protein; Cooking loss and drip loss are expressed as percentages. SEM, standard error of the mean.

**Table 8:** Effects of dietary ME and CP levels on serum biochemical indices of goslings at 63 d of age.

Items	ME (MJ/kg)	CP (%)	TP (g/L)	ALB (g/L)	GLB (g/L)	A/G	GLU (mmol/L)	TC (mmol/L)	TG (mmol/L)	HDL-C (mmol/L)	LDL-C (mmol/L)	Urea (μmol/L)
NPNE	11.20	16	45.53	15.60	29.93	0.530	11.62 ^a^	3.918	2.778	1.600	1.465	1.005
LPNE		14	39.40	14.10	25.30	0.558	14.01 ^b^	3.938	2.527	1.863	1.307	0.977
NPHE	11.65	16	42.60	15.23	27.37	0.562	12.36 ^ab^	4.030	2.565	1.842	1.47	0.993
LPHE		14	42.85	14.63	28.22	0.523	11.10 ^a^	3.820	1.918	1.727	1.405	0.993
ME	11.20		42.47	14.85	27.62	0.544	12.82 ^b^	3.928	2.653 ^a^	1.732	1.386	0.991
	11.65		42.73	14.93	27.79	0.543	11.73 ^a^	3.925	2.242 ^b^	1.784	1.438	0.993
CP		16	44.07	15.42 ^a^	28.65	0.546	11.99	3.974	2.672 ^a^	1.721	1.468	0.999
		14	41.13	14.37 ^b^	26.76	0.541	12.55	3.879	2.223 ^b^	1.795	1.356	0.985
SEM			0.960	0.238	0.774	0.010	0.326	0.08	0.093	0.051	0.039	0.023
*p* value	ME		0.888	0.852	0.907	0.936	0.040	0.984	0.008	0.599	0.517	0.959
	CP		0.121	0.027	0.217	0.810	0.272	0.578	0.004	0.459	0.169	0.772
	ME × CP		0.094	0.319	0.079	0.120	0.001	0.502	0.169	0.068	0.558	0.772

NPNE, normal-protein normal-energy; LPNE, low-protein normal-energy; NPHE, normal-protein high-energy; LPHE, low-protein high-energy. ME, metabolizable energy; CP, crude protein; TP, total protein; ALB, albumin; GLB, globulin; A/G, albumin-to-globulin ratio; GLU, glucose; TC, total cholesterol; TG, triglycerides; HDL-C, high-density lipoprotein cholesterol; LDL-C, low-density lipoprotein cholesterol. SEM, standard error of the mean. *p*-values were obtained by two-way ANOVA (GLM) with dietary ME, CP, and their interaction (ME × CP) as fixed effects. When a significant interaction or treatment effect was detected, means were separated using the least significant difference (LSD) test. Means within a column with different superscript letters differ significantly (*p* < 0.05).

**Table 9:** Effects of dietary ME and CP levels on taste attributes of gosling meat at 63 d of age.

Items	ME (MJ/kg)	CP (%)	Sourness	Bitterness	Astringency	Aftertaste-B	Aftertaste-A	Umami	Richness	Saltness	Sweetness
NPNE	11.20	16	−37.32	3.00	−2.52	−1.27	−0.71	10.78	2.07	−4.00	−1.99
LPNE		14	−38.29	3.05	−2.66	−1.26	−0.67	10.80	2.04	−3.97	−1.93
NPHE	11.65	16	−37.80	3.25	−2.53	−1.17	−0.70	10.83	2.01	−4.00	−1.93
LPHE		14	−37.91	3.27	−2.28	−0.96	−0.50	10.09	2.30	−4.27	−1.57
ME	11.20		−37.56	3.03	−2.53	−1.27	−0.71	10.79	2.06	−3.99	−1.96
	11.65		−38.10	3.26	−2.47	−1.06	−0.59	10.46	2.16	−4.14	−1.75
CP		16	−37.80	3.13	−2.59	−1.22	−0.69	10.81	2.04	−4.00	−1.96
		14	−37.85	3.16	−2.40	−1.11	−0.60	10.44	2.17	−4.12	−1.75
SEM			0.337	0.099	0.110	0.082	0.064	0.276	0.116	0.125	0.181
*p* value	ME		0.279	0.311	0.716	0.107	0.211	0.409	0.537	0.419	0.431
	CP		0.918	0.809	0.250	0.361	0.343	0.370	0.447	0.497	0.433
	ME × CP		0.388	0.572	0.233	0.386	0.351	0.346	0.349	0.411	0.581

NPNE, normal-protein normal-energy; LPNE, low-protein normal-energy; NPHE, normal-protein high-energy; LPHE, low-protein high-energy. ME, metabolizable energy; CP, crude protein; Taste attributes were evaluated instrumentally. SEM, standard error of the mean.

## Data Availability

The original contributions presented in this study are included in the article/Supplementary Materials; further inquiries can be directed to the corresponding author.

## Supplementary Materials

The following supporting information can be downloaded at: https://www.mdpi.com/article/10.3390/foods15061060/s1, The ARRIVE guidelines 2.0: author checklist.
